# Dietary supplement use among undergraduate male students in health and non-health cluster colleges of a public-sector university in Dammam, Saudi Arabia

**DOI:** 10.1186/s12906-018-2332-4

**Published:** 2018-10-01

**Authors:** Atta Abbas Naqvi, Rizwan Ahmad, Abdullah Abdul Wahid Elewi, Ayman Hussain AlAwa, Moayed Jafar Alasiri

**Affiliations:** 1 Department of Pharmacy Practice, College of Clinical Pharmacy, Imam Abdulrahman Bin Faisal University, Dammam, 31441 Saudi Arabia; 2 Natural Products and Alternative Medicines, College of Clinical Pharmacy, Imam Abdulrahman Bin Faisal University, Dammam, 31441 Saudi Arabia; 3 College of Clinical Pharmacy, Imam Abdulrahman Bin Faisal University, Dammam, 31441 Saudi Arabia

**Keywords:** Dietary supplement, Undergraduate students, Prevalence, Opinions, Attitudes, Cost, Saudi Arabia

## Abstract

**Background:**

Dietary supplements (DS) are nutraceuticals that improve overall health and well-being of an individual as well as reduce the risk of diseases. Evidence indicates a rising prevalence of these products worldwide especially among college students. Studies have reported an increasing use of supplements among Saudi students. However, the scope of those researches was limited to prevalence data. Hence, the aim of our study was to document the prevalence, opinions, attitudes, reasons for use and monthly cost attributed to dietary supplement use.

**Methods:**

A 3-month cross-sectional study was conducted to evaluate use of dietary supplement among health and non-health college students at a public-sector university in Dammam city, Saudi Arabia. It was conducted using Arabic version of the Dietary supplement questionnaire (DSQ-A). A total of 469 male students responded to the survey giving a response rate of 93.8%. The students were from ten colleges of the university. The data was analyzed by SPSS version 22. The study was approved by Institutional Review Board of Imam Abdulrahman Bin Faisal University, Dammam, Saudi Arabia (IRB-UGS-2018-05-074).

**Results:**

The overall prevalence of dietary supplement use in the university was 29.42%. In health cluster colleges, it was reported at 35.91% while in non-health cluster college it was 23.69%. Maintaining general health and well-being was the most common reason for use. Prevalence of multivitamins and whey proteins was approximately 23%. Average monthly cost of supplement was SAR 278.92 (USD 74.39). Cost was positively correlated (ρ = 0.305) with satisfaction score. Students preferred brand products (16.4%). 41.4% students opined that DS may prevent chronic illness if used regularly and agreed that they are good for health. Majority of students (65%) recommended DS use only upon physician’s recommendation. College clusters and study-year was associated (*p*-value< 0.01) with students’ opinion. Students in health cluster colleges were more likely to recommend supplements (OR 3.715, *p*-value< 0.0001).

**Conclusion:**

Prevalence of dietary supplement use was lower than other local and international university students. Health cluster colleges had higher prevalence as compared to non-health cluster colleges. Multivitamins and whey protein were the most commonly used types of DS. Students preferred brand products, had positive opinions and attitudes towards dietary supplement. However, they recommended supplements use to others only upon a physician’s recommendation.

**Electronic supplementary material:**

The online version of this article (10.1186/s12906-018-2332-4) contains supplementary material, which is available to authorized users.

## Background

Appropriate nutrition is essential for proper development and maintenance of the human body. Dietary supplement (DS) improves general health and well-being when used as recommended [1]. It includes multivitamins, minerals and, natural products extracts [2]. The use of DS is quite common in the developed world [3]. According to the 3rd National Health and Nutrition Examination Survey (NHANES), the overall prevalence of dietary supplement use in the United States (US) was 40% [3]. Evidence indicates that individuals may use a single or a combination of different dietary supplements as means to improve their nutrition intake, maintaining general health and well-being, as well as reduce risk of diseases [3]. Conversely, studies have reported adverse drug events and mortalities because of over dose, drug-drug and drug-disease interactions associated with supplement use [4]. For instance, excessive use of cholecalciferol, i.e., vitamin D, may adversely affect soft tissues and kidney functions [5]. Supplements may also interact with drugs for managing cardiovascular illnesses [6, 7].

Studies have reported a higher consumption of supplements in subgroups as compared to overall population and, several demographic determinants such as higher education, attitudes, etc., have been reported to be associated with a greater likelihood of dietary use [3, 8, 9]. Literature highlights that more than half (66%) of college students in the US used dietary supplements and students with a health study background used DS more regularly than students belonging to non-health study field [1, 10, 11].

The monetary value of dietary supplements in 2014 amounted to United States dollar (USD) 165.62 billion and was rising with a compound annual growth rate (CAGR) of 7.3%. The projected market worth is expected to touch USD 278.96 billion by 2021. Asia Pacific region has emerged as the second largest market for dietary supplements after US and Canada [12]. Saudi Arabia is the biggest market for dietary supplements in the Middle East region as DS accounts for 4% of total pharmaceuticals sold in the country with an estimated worth of USD 2 billion [13, 14].

Dietary supplements may be marketed as brand or generic products. A brand is a pharmaceutical product that is developed by a pharmaceutical company after years of research and financial investment. These pharmaceutical products are legally protected by patents granted from health regulatory agencies for several years. The patent gives the manufacturer the sole legal right to sell the product for the stated period of patent. After the expiry of the patent, other pharmaceutical firms may manufacture the product after approval from the health regulatory agency. These products are termed as generic. Since generic products utilize the research of the parent brand drug, they do not require investment of huge finances in research and therefore, are cheaper than their brand counterparts. However, studies have reported that despite same product quality, brands may be preferred over generics due to a positive patient perception [15].

College students studying in Saudi Arabia were more likely to use DS as compared to general population [10]. Although, a few studies reported that the use of supplements had increased in general population as well as undergraduate students of Saudi Arabia. However, the findings were limited to prevalence data [14, 16]. There is a scarcity of data that reports common supplements used by Saudi students. Moreover, the reasons as well as attitudes towards DS use have not been studied before. A study by Albusalih et al., conducted at this venue reported an increasing use of dietary supplement among students [16]. However, it established a mere prevalence of multivitamins. Hence, the aim of this study was to report the use of dietary supplements in students including its prevalence data, i.e., overall, college-wise and study year-wise prevalence as well as individual prevalence of each supplement. In addition, students’ opinion, attitudes, reasons for use as well as monthly cost attributed to dietary supplement use, was documented.

## Methods

A cross-sectional study was conducted in undergraduate students studying in health and non-health cluster colleges at Imam Abdulrahman Bin Faisal University located in Dammam, Saudi Arabia.

### Operational definitions

#### Dietary supplements

Dietary supplements are nutraceuticals and natural products extracts, that are available without prescription and are used by individuals in recommended dose for improving performance, general health and well-being as well as reducing the risk of diseases [1].

#### Dietary supplement prevalence (point prevalence)

The regular use of dietary supplements in a population over a defined time-period [1].

#### Brand and generic pharmaceutical products

A brand is a pharmaceutical product that is developed by a pharmaceutical company after years of research and financial investment. They are legally protected by patents granted from health regulatory agencies for several years that gives the manufacturer the sole legal right to sell the product for the stated period of patent. After the expiry of the patent, other pharmaceutical firms may manufacture the product after approval from the health regulatory agency. These products are termed as generics and are cheaper than brands because they utilize the research of the parent brand drug and do not require investment of huge finances in research [15].

#### Target population and inclusion criteria

The study included male students studying in health and non-health colleges affiliated to IAU who were willing to participate. Health cluster included four colleges namely clinical pharmacy, medicine, nursing and applied medical science. Non-health cluster included six colleges namely sharia and law, architecture and planning, engineering, applied studies and community service, business and, science. Female students, students who had graduated from the university and non-consenting students were excluded. Female students could not be included as male and female students were segregated and studied in different campuses with separate and independent administration. The study included only male investigators and it was not possible to conduct the study in female campuses. This is mentioned as a limitation of our study.

#### Sample size and procedure

We employed purposive sampling methodology and selected most convenient time such as prayer and lunch breaks to approach students. Students from different health and non-health cluster colleges of university were invited to participate. Sample size was calculated based on number of undergraduate students studying in Saudi universities. According to official figures, there were 1597 students enrolled at IAU [17]. This figure was identified as target population. Sample size was calculated from online calculator [18]. The sample size calculated was 310. Our study gathered data from 469 students which was more than the required sample size.

#### Research instrument and translation process

A survey questionnaire developed by Naqvi and colleagues, known as the Dietary supplement questionnaire (DSQ) [1], was used after its translation into Arabic language that is the native language of Saudi students. The questionnaire contained multiple choice questions, few open-ended questions and a numeric rating scale.

The DSQ includes questions related to the demographic information of respondents such as age, college, year of study, residence status, number of siblings, presence of any major illnesses and if they had used a dietary supplement in the last month. Furthermore, the respondents were asked about the type of dietary supplement they used, what commercial product did they use, i.e., imported brand from foreign countries or a locally produced generic product, the reason for taking supplements and monthly cost attributed to its use. The respondents were also asked if they suffered from any adverse drug event that was related to their supplement use. The respondents were also inquired about their source of information and opinions about supplements. They were asked if they believed that dietary supplements were good for health and whether they would personally recommend supplements to others. Finally, they were asked to rate their satisfaction with supplement use on a rating scale [1].

The translation was carried out considering standard guidelines [19, 20]. The translation process included three Arabic speaking pharmacists whose second language was English. The initial translation was conducted in supervision of the inventors of DSQ to elaborate and clarify meaning in any DSQ item. The initial draft was reviewed by four academic professors specialized in medicine, pharmacy and physics whose first language was Arabic and second language was English. This was done to check for adaptability of the Arabic version (DSQ-A) for respondents belonging to health and non-health background. The three reviewers were kept unaware of the purpose of study to have a balanced review of the tool. DSQ-A was back translated by an academic professor of ethics with same language expertise in collaboration with tool inventors. The face validity and content validity were established and the Arabic version of DSQ was deemed fit to use at this point. The questionnaire is available as an Additional file 1.

#### Pilot study and acceptability of the DSQ-A

DSQ-A was subjected to pilot study in 100 students belonging to different colleges of the university. A total of 92 responses were received giving a response rate of 92%. No difficulty in understanding of the questionnaire was observed. The DSQ-A was deemed fit to use at this point.

#### Data analysis

The data was entered and analyzed using statistical software IBM SPSS version 22. Prevalence was calculated using Medcalc. Frequency counts (N), percentages (%) and descriptive statistics such as mean (X) and standard deviation (SD) were used. Prevalence was expressed in percentage (%) and 95% confidence intervals. Inferential statistics such as chi square (χ2) test to observe associations between student characteristics, i.e., independent variables (IV) and DS study variables, i.e., dependent variables (DV) were performed. Spearman’s rank correlations (ρ) was employed to document any correlation among IV and DV. Regression analysis was conducted to report any predictors of DS use.

#### Ethics approval and consent

The study was approved by the Institutional Review Board of Imam Abdulrahman Bin Faisal University (IRB-UGS-2018-05-074). Students were informed about the study and its objectives. Students who consented to participate in the study were included. The participation was voluntary without any incentive. An informed written consent was sought from students before handing them the questionnaire. Those who consented to participate in the study were handed DSQ-A.

## Results

Of total 500 students who were approached, 469 students responded to the survey giving a response rate of 93.8%. The study included students from all study years. The mean age of the students was 21 years (X = 20.96, SD = 1.66). Majority of the students (*N* = 395, 84.2%) lived with their families and had 3–5 siblings (*N* = 262, 55.9%). Few students (*N* = 49, 10.4%) suffered from major illnesses. Of total 49 patients considered as 100% who suffered from illnesses, the proportions of patients were; hypertension (*N* = 3, 6.1%), diabetes mellitus (*N* = 6, 12.2%), sickle cell anemia (*N* = 7, 14.3%), thalassemia (*N* = 1, 2%), glucose 6 – phosphate dehydrogenase (G6PD) (*N* = 17, 34.7%), asthma (*N* = 6, 12.2%), rheumatoid arthritis (*N* = 1, 2%), psoriasis (*N* = 2, 4%), gastro esophageal reflux disorder (GERD) (*N* = 1, 2%), migraine (*N* = 2, 4%), obesity (*N* = 2, 4%) and epilepsy (*N* = 1, 2%). The summary of student information is presented in Table 1.

**Table 1:** Student characteristics

	N	%
College		
Pharmacy	70	14.9
Medicine	92	19.6
Nursing	22	4.7
Sharia and law	39	8.3
Applied medical sciences	36	7.7
Architecture and planning	57	12.2
Engineering	36	7.7
Applied studies and community service	27	5.8
Business	43	9.2
Science	47	10
Study year		
Prep year	84	17.9
2nd year	138	29.4
3rd year	92	19.6
4th year	59	12.6
5th year	89	19
6th year	7	1.5
Residence		
Living with family	395	84.2
Living alone (University accommodation)	74	15.8
Siblings		
Between 1 and 2 siblings	49	10.4
Between 3 and 5 siblings	262	55.9
Between 6 and 8 siblings	102	21.7
More than 8 siblings	50	10.7
No siblings	6	1.3
Any major illness		
Do not suffer from any illness	420	89.6
Suffer from a major illness	49	10.4
DS use in the last month		
Daily	62	13.2
Weekly	24	5.1
Once a month	20	4.3
Never	300	64
Not sure	63	13.4

### Types and prevalence of dietary supplement use

Multivitamins were used alone or in combination by a tenth proportion of students, i.e., 9–9.4%. The overall prevalence of dietary supplement use was 29.4%. Health cluster colleges had an overall prevalence of 35.9% while non-health cluster college reported a prevalence of 23.7%. The summary of prevalence by group and individual colleges as well as study year is presented in Table 2.

**Table 2:** Types and prevalence of dietary supplement used

Types of dietary supplements used	Alone	As combination
Multivitamins	44 (9.4)	42 (9)
Gingko biloba	1 (0.2)	–
Ginseng	–	3 (0.6)
Glucosamine/omega 3 FA	3 (0.6)	32 (6.8)
Whey protein	38 (8.1)	38 (8.1)
Calcium	1 (0.2)	–
Creatine phosphate	–	1 (0.2)
More than one supplement	64 (13.6)	–
Not applicable	318 (67.8)	–
Cluster-wise prevalence	Prevalence (%)	95% confidence interval
All colleges	29.4	25.3–33.8%
Health cluster colleges	35.9	29.6–42.6%
Non-health cluster colleges	23.7	18.5–29.5%
College-wise prevalence		
Pharmacy	34.3	23.4–46.6%
Medicine	39.1	29.1–49.9%
Nursing	18.2	5.2–40.3%
Applied Medical Science	40	23.9–57.9%
Sharia and law	12.8	4.3–27.4%
Architecture and planning	14	6.6–25.8%
Engineering	41.7	25.5–59.2%
Applied studies and community service	18.5	6.3–38.1%
Business	37.2	23–53.3%
Science	19.2	9.2–33.3%
Study year-wise prevalence		
Prep year	16.7	9.4–26.4%
2nd year	24.5	17.6–32.5%
3rd year	29.4	20.3–39.8%
4th year	37.3	25–50.6%
5th year	42.7	32.3–53.6%
6th year	42.9	9.9–81.6%

The college-wise and study year-wise prevalence of multivitamins, glucosamine/omega 3 Fatty Acids (FA), whey protein, ginseng and gingko biloba was documented. The prevalence of ginseng was reported at 2.9% (0.4–9.9 for 95% CI) from pharmacy college. It was also reported at 1.1% (0.03–5.6 for 95% CI) and 1.7% (0.04–8.9 for 95% CI) in 3rd and 4th year students respectively. The prevalence of gingko biloba was reported in pharmacy college at 1.4 (0.04–7.7 for 95% CI) and at 1.7% (0.04–8.9 for 95% CI) in 4th year students respectively. The college and study year-wise prevalence are tabulated in Table 3.

**Table 3:** College and study year-wise prevalence of multivitamins, glucosamine/Omega 3 FA and whey protein

	Prevalence % (95% CI)		
	Multivitamins	Glucosamine/Omega 3 FA	Whey protein
Colleges			
Pharmacy	18.6 (10.3–29.7)	10 (4.1–19.5)	22.9 (13.7–34.5)
Medicine	21.7 (13.8–31.6)	6.5 (2.4–13.7)	25 (16.6–35.1)
Nursing	9.1 (1.1–29.2)	9.1 (1.1–29.2)	12.5 (2.7–32.4)
Applied Medical Science	18.2 (8.2–32.8)	10 (2.8–23.7)	16.3 (6.8–30.7)
Sharia and law	9.3 (2.6–22.2)	2.5 (0.1–13.2)	4.9 (0.6–16.5)
Architecture and planning	10.9 (4.5–21.3)	9.5 (3.6–19.6)	13.6 (6.4–24.3)
Engineering	18.2 (8.2–32.7)	12.2 (4.1–26.2)	12.2 (4.1–26.2)
Applied studies and community service	10 (2.1–26.5)	–	12.9 (3.6–29.8)
Business	23.2 (3–36.4)	2.3 (0.1–12)	17.3 (8.2–3.3)
Science	16.1 (7.6–28.3)	6 (1.3–16.6)	9.6 (3.2–21)
Study year			
Prep year	15.2 (8.7–23.8)	3.5 (0.7–9.8)	6.7 (2.5–14)
2nd year	9.8 (5.6–15.7)	7.4 (3.7–12.8)	10.4 (6.1–16.3)
3rd year	17.8 (11.3–26.2)	8.9 (4.2–16.2)	13.2 (7.4–21.2)
4th year	21.3 (12.7–32.3)	6.4 (1.8–15.5)	19.2 (10.9–30.1)
5th year	16.8 (10.3–25.3)	6.3 (2.4–13.2)	23.4 (16.5–32.7)
6th year	30 (6.7–65.2)	22.2 (2.8–60.1)	22.2 (2.8–60.1)

### Cost of dietary supplement per month

The average monthly cost attributed to dietary supplement use was reported at SAR 278.92 (USD 74.39) however, the median cost reported was SAR 220 (USD 58.67). Minimum cost incurred was reported at SAR 5 (USD 1.33) and maximum cost incurred on DS use per month was SAR 2170 (USD 578.74).

### Reasons and types of dietary supplements used

Most students did not use DS (318, 67.8%). Almost a fifth proportion of students (*N* = 82, 17.5%) selected more than one reason for using dietary supplement.

### Students’ experience from DS use

Some students (*N* = 77, 16.4%) used a brand product and a few (*N* = 51, 10.9%) did not know if it was a brand or generic product. A third of students (*N* = 142, 30.2%) did not suffer from an adverse reaction to dietary supplement.

### Opinion and attitudes towards DS

More than 40% of students (*N* = 194, 41.4%) opined that DS may prevent chronic illness if used regularly followed by similar number of students (*N* = 185, 39.4%) who considered DS as harmless. Slightly less than half of students (*N* = 224, 47.8%) agreed to the notion that DS are good for health and more than half (*N* = 305, 65%) recommended use of DS to others, only upon doctor’s recommendation. Student rated their satisfaction with DS use on a scale of 0 (worst) to 5 (best). The average satisfaction score was reported to be almost 3 (X = 2.89, SD = 1.25). The details are presented in Table 4.

**Table 4:** Reasons for use, experience with DS, opinions and attitudes towards dietary supplements

Reasons for use	Sample (N)	Percentage (%)
General Health and Well Being	22, 31^a^	4.7, 6.6^a^
Boost immunity	2, 27^a^	0.4, 5.8^a^
Weight gain	2, 18^a^	0.4, 3.8^a^
Doctor’s recommendation	7, 17^a^	1.5, 3.6^a^
Enhance memory	3, 16^a^	3.4, 0.6^a^
Increase performance/sports	5, 24^a^	1.1, 5.1^a^
Increase endurance/body building	12, 47^a^	2.6, 10^a^
Malnutrition	9. 35^a^	1.9, 7.5^a^
Energy source	7, 29^a^	1.5, 6.2^a^
More than one reason	82	17.5
Not applicable	318	67.8
Experience with DS use in last month		
Pharmaceutical category used		
Generic	12	2.6
Brand	77	16.4
Both	11	2.3
I do not know	51	10.9
Not applicable	318	67.8
Adverse reactions to dietary supplements		
I suffered from an adverse reaction	1	0.2
I suffered from an adverse reaction but sure if it was related to DS use	8	1.7
No, I did not suffer from any adverse effect	142	30.2
Not applicable to me as I did not use any supplements	318	67.8
Opinion and attitudes		
Opinion about dietary supplements		
Necessary for all ages	49	10.4
They are harmless	185	39.4
Regular use of dietary supplement prevents chronic disease	194	41.4
Dietary supplements may prevent cancer	26	5.5
No opinion	15	3.2
Dietary supplements are good for health		
Agree	224	47.8
I don’t know	173	36.9
Disagree	72	15.4
Do you personally recommend use of dietary supplements to others		
Yes, I always recommend	66	14.1
Yes, only when doctors recommend	305	65
Not at all	98	20.9

Legend (^a^) = selected in combination with others

### Association and correlation of independent and dependent variables

The dependent variable (DV) of student opinion towards dietary supplements, was significantly associated with; college (*p*-value< 0.01), college type (*p*-value< 0.01) and study year (*p*-value< 0.001). However, there was no statistical significance between the variable of student opinion and demographic information, i.e., siblings (*p*-value> 0.05). Moreover, the association between student opinion and illness profile was insignificant (*p*-value> 0.05). The cross tabulation is presented in Table 5. The values presented in brackets are expected counts.

**Table 5:** Cross tabulation between students’ demographic characteristics and opinion

Independent Variable	Dietary supplements are good for health			*P*-value
	Agree	Do not know	Disagree	
College				*< 0.01*
Clinical pharmacy	49 (33.4)	17 (25.8)	4 (10.7)	
Medicine	62 (43.9)	23 (33.9)	7 (14.1)	
Nursing	7 (10.5)	9 (8.1)	6 (3.4)	
Applied medical sciences	12 (17.2)	14 (13.3)	10 (5.5)	
Sharia and law	10 (18.6)	23 (14.4)	6 (6)	
Architecture and planning	15 (27.2)	32 (21)	10 (8.8)	
Engineering	21 (17.2)	9 (13.3)	6 (5.5)	
Applied studies and community service	7 (12.9)	13 (10)	7 (4.1)	
Business	27 (20.5)	11 (15.9)	5 (6.6)	
Science	14 (22.4)	22 (17.3)	11 (7.2)	
College cluster				*< 0.001*
Health	130 (105.1)	63 (81.2)	27 (33.8)	
Non-health	94 (118.9)	110 (91.8)	45 (38.2)	
Study year				*< 0.001*
Prep Year	26 (40.1)	39 (31)	19 (12.9)	
2nd Year	52 (65.9)	63 (50.9)	23 (21.2)	
3rd Year	47 (43.9)	32 (33.9)	13 (14.1)	
4th Year	35 (28.2)	17 (21.8)	7 (9.1)	
5th Year	61 (42.5)	19 (32.8)	9 (13.7)	
6th Year	3 (3.3)	3 (2.6)	1 (1.1)	
Siblings				*> 0.05*
Between 1 and 2 siblings	28 (23.4)	14 (18.1)	7 (7.5)	
Between 3 and 5 siblings	120 (125.1)	103 (96.6)	39 (40.2)	
Between 6 and 8 siblings	54 (48.7)	35 (37.6)	13 (15.7)	
More than 8 siblings	22 (23.9)	17 (18.4)	11 (7.7)	
No siblings	0 (2.9)	4 (2.2)	2 (0.9)	
Any major illness				*> 0.05*
Do not suffer from any illness	198 (200.6)	156 (154.9)	66 (64.5)	
Suffer from a major illness	26 (23.4)	17 (18.1)	6 (7.5)	

**Fig. 1:**
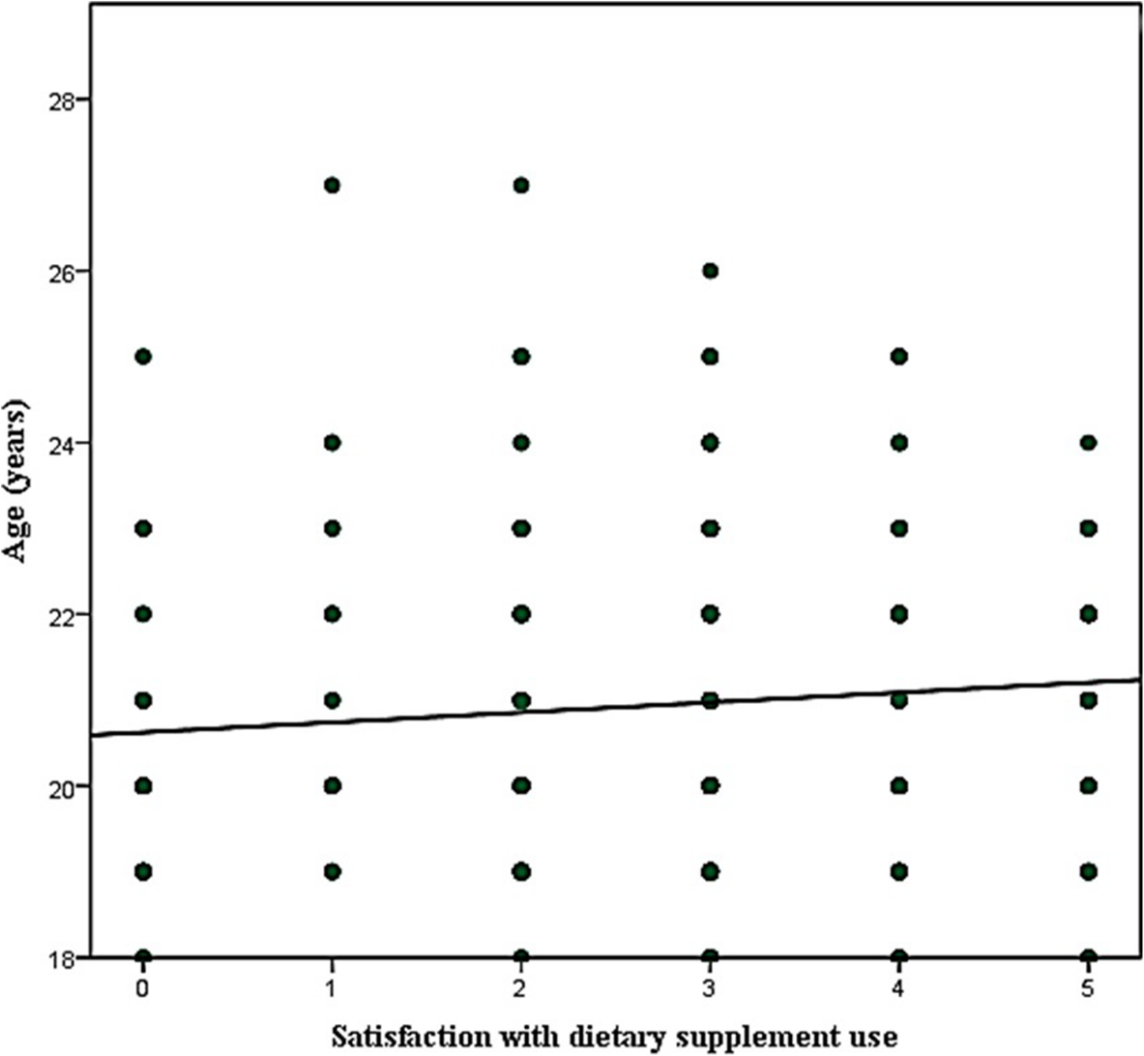
Correlation of student age with satisfaction score

**Fig. 2:**
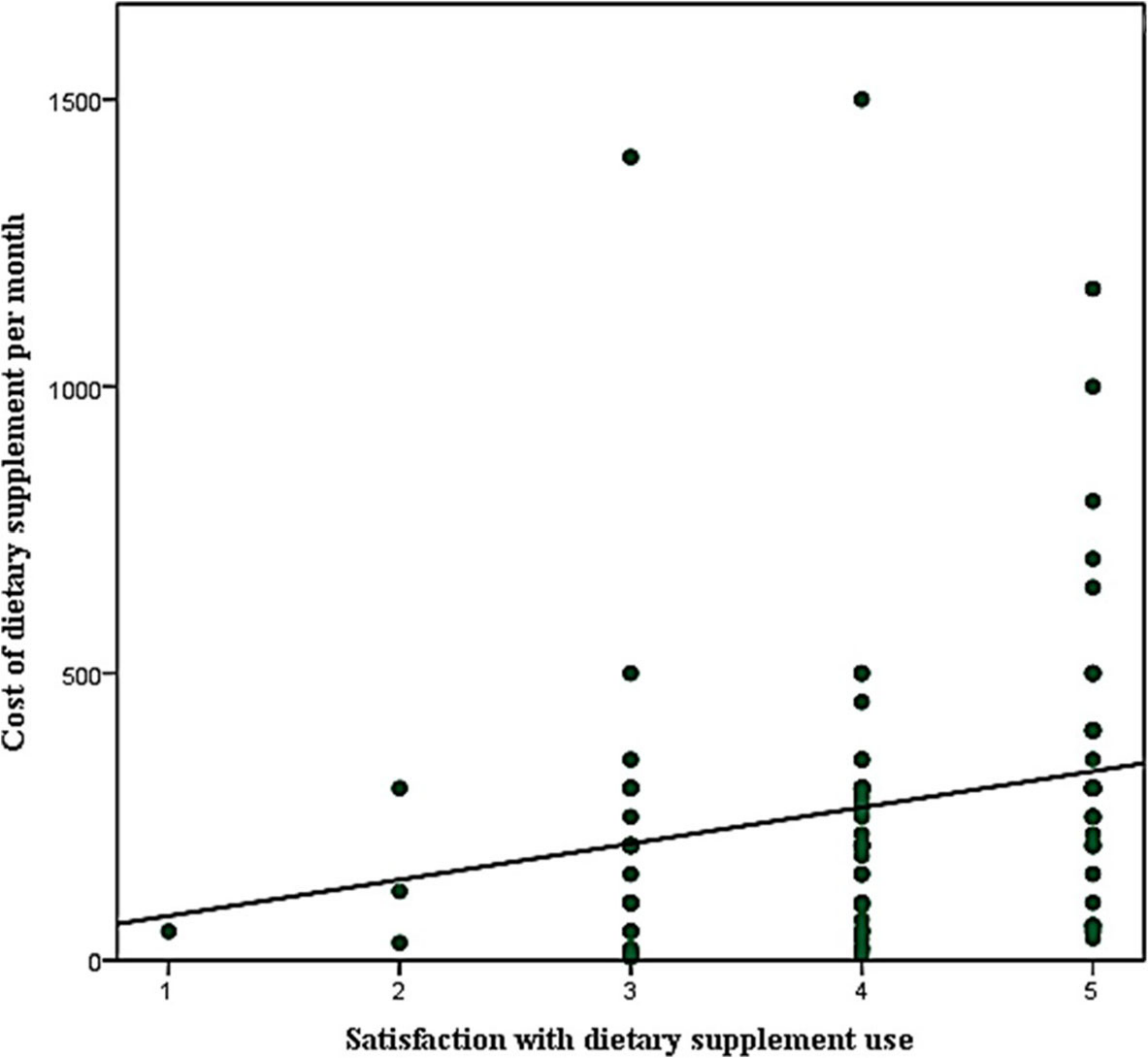
Correlation of cost of dietary supplement with satisfaction score

The IV of age and dietary supplement cost were positively correlated with satisfaction score (ρ = 0.12 and ρ = 0.305 respectively) and the correlation was significant at *p*-value< 0.01 (Figs. 1 and 2).

Multinomial logistic regression (MLR) was used to interpret the odds ratio for dependent variable (DV) of, ‘encouraging dietary supplement use’. The discrete independent variable (IV) of, ‘age’ was considered as covariate and categorical IV of, ‘college type’ was fixed. Student attitudes towards encouraging DS use was taken as DV. The parameter of IV, i.e., ‘non-health cluster college’, was redundant and therefore, set to zero (0). Parameter of DV, i.e., ‘not at all’, was considered as reference category. The regression analysis reported that keeping the categorical IV of ‘college type’ as constant, the odds of, ‘always recommending DS use’, increases with every year-wise increase in age (OR 1.303, *p*-value< 0.01). Similarly, keeping the IV of ‘age’ as constant, students studying in ‘health cluster colleges’ were more likely to, ‘recommend DS use’ as compared to students studying in ‘non-health cluster colleges’ (OR 3.715, *p*-value< 0.0001). The regression analysis is tabulated in Table 6.

**Table 6:** Multinomial logistic regression analysis

Encourage dietary supplement use?		Coefficient	Odds ratio	95% Confidence interval	
				Lower bound	Upper bound
Yes, I always recommend	Age	0.265	1.303	1.080	1.572
	Health cluster college	1.312	3.715	1.899	7.266
	Non-health cluster college	0^a^	–	–	–
Yes, only when doctor recommends	Age	0.068	1.07	0.93	1.231
	Health cluster college	1.133	3.104	1.868	5.156
	Non-health cluster college	0^a^	–	–	–

^a^Reference category

## Discussion

Dietary supplement use has been previously reported from this university by Albusalih et al., that highlighted the need to conduct a full-scale study to investigate their use including prevalence, cost attributed to DS use and students’ attitude and opinion towards it [16]. This study was conducted in ten colleges of the university. The sample size was 310 for 95% confidence interval (CI) however, we focused on gathering sample to satisfy 99% CI. The extensiveness of data collection in ten colleges, large sample size and a high response rate are major strengths of this study.

The average age of students was 21 years and most students lived with their families. Few students had major illnesses, i.e., cardiovascular diseases, endocrine and blood disorders, musculoskeletal diseases, gastrointestinal and central nervous system disorders. Previous studies conducted in this population have also reported similar student health profile [16, 21–23].

The overall prevalence of DS use at the university was 29.42%. This reiterates the findings of Albusalih et al., that reported a 30% prevalence of the same [16]. Though, that figure was reported in male and female students cumulatively. Our results are in the range of 26.31–35% as reported by Albusalih et al., [16]. Naqvi and colleagues reported a prevalence of dietary supplement use at 51% in male undergraduate pharmacy students of Pakistani universities [1]. Our finding was much lower than figures reported from American, Nigerian and South African university students [1, 14, 24, 25]. However, it was higher than that reported in Japanese students [26]. Our study further documented college-wise and study-year wise prevalence data as well. We tested the hypothesis that health students use such products more than non-health students. Studies have reported that students with a health background tend to use supplements more than their counterparts which could be due to better awareness and education that may shape their opinions in favour of using supplements [26, 27]. The prevalence of DS use in health cluster colleges was reported at 35.91% while it was 23.69% in non-health cluster. Similarly, highest prevalence of DS use was reported from applied medical science, medicine and pharmacy colleges. Students of engineering college, i.e., a non-health college, reported prevalence similar to that reported in students of applied medical science college. This reiterates the findings of Kobayashi et al., that prevalence was higher in students with health background compared to those with a non-health background [11, 26]. An inclining trend was evident while investigating prevalence data based on study year. This occurrence has been reported previously among university student in Japan. Hence, our findings are consistent with Kobayashi and colleagues [26].

Prevalence of some dietary supplements that were observed to be common amongst students in all colleges was also determined. These included multivitamins, glucosamine/Omega 3 fatty acids, whey protein, ginseng and gingko biloba extracts. Most notable figures were documented for whey protein use. The prevalence remained consistent throughout all colleges. Evidence indicates that protein supplementation is quite common among male university students as it improves physical performance [28, 29]. Multivitamin use prevailed in all colleges with highest prevalence in business college, i.e., around 23% and lowest in nursing college, i.e., 9.1%.

The use of glucosamine/Omega 3 fatty acids (FA) was more widespread in health cluster as compared to non-health cluster. Omega 3 FA may improve sleep quality, reduce depression and anxiety among students [30, 31]. Abbas et al., have reported that anxiety and depression due to academic studies may prevail in undergraduate students [32–34]. Prohaska reported improvement in sleep efficiency by omega 3 FA supplementation in clinically depressed undergraduate students [31]. Studies by Al Rasheed et al., and Al-Shagawi et al., have reported high academic stress and anxiety in Saudi undergraduate students studying medicine and allied health subjects [21, 22]. Hence, its prevalent use could be due to the stress and anxiety among health track students. Use of omega 3 fatty acids has been reported from other colleges in Saudi Arabia as well [14]. However, we could not conclude that high academic stress, depression and anxiety was the reason behind prevalent use of omega 3 FA in our sample.

The use of ginseng and gingko biloba was reported in very low percentages mainly from 3rd and 4th year pharmacy students. This is relevant as pharmacy students study natural products and alternative medicines as well as pharmacognosy courses that may increase their knowledge and awareness of natural products [23]. Use of ginseng and gingko biloba has also been reported from other Saudi universities [14].

Most common reason mentioned for using DS was maintenance of general health and well-being. Students in Pakistani universities mentioned physician’s advice as most common reason to use dietary supplement [1]. Furthermore, multivitamins were the most common type of DS used alone, as well as in combination. This occurrence has been reported by studies conducted in local and international academia [8, 14, 16, 26].

The average monthly cost attributed to dietary supplement use was reported at SAR 278.92, i.e., USD 74.39. No studies have been conducted in Saudi universities till date, that document cost attributed to DS use among students. A novel study conducted by Naqvi et al., in Pakistani universities reported average DS cost at USD 13.5 [1]. Hence, our findings highlight a relatively high spending trend among Saudi students. However, this may not be definite as there may be other determinants such as socio-economic status that may affect spending on supplements. We found that students preferred a brand over generic dietary supplement. This may be based on individual buying preference. Studies have demonstrated that an individual may prefer brands as brand products are believed to have better quality as compared to generics [15, 28]. Dwyer and colleagues have reported that poor quality of dietary supplements have resulted in adverse drug events (ADEs) and mortality [6]. Moreover, pharmaceutical packaging may also affect consumers’ preferences. Sabah and colleagues mentioned un-attractive packaging as a reason for low preference of generic products in Pakistan [35]. The occurrence of a significantly strong correlation between cost of DS and satisfaction score may explain the buying preference from another dimension. This correlation implied that students appeared more satisfied with expensive supplements. This occurrence has been reported by Jamshed and colleagues from Pakistan’s health sector as well [36]. Studies report that brands are generally expensive than generics [15, 36].

Majority of the students opined that regular use of supplements prevented chronic diseases and agreed to the idea that DS were good for health. This was significantly associated with demographic variables of college, college cluster and study year. Students belonging to health cluster colleges agreed that DS were good for health however, responses from non-health track students swayed between uncertainty and disagreement. Similarly, students in advance study years agreed with the notion. These findings agree with Kobayashi and colleagues [26]. Another confirmation of this phenomenon was the occurrence of significant positive correlation between age of students and DS satisfaction score. Most students mentioned that they would recommend DS only upon a physician’s recommendation. This finding contradicts the results of El Khoury et al., i.e., Lebanese students recommended use of supplements on relatives, friends and peers’ advice [37]. However, there is a plethora of evidence that indicates that dietary supplements may be harmful if not taken in recommended dose [4]. In some cases, over usage of multivitamins may exceed the permissible limits of certain vitamins such as cholecalciferol that may result in adverse effects [5, 6, 38].

## Conclusion

The prevalence of dietary supplement was lower than other local and international academic institutions. Prevalence was higher in health cluster colleges as compared to non-health cluster colleges. Multivitamins were most commonly used dietary supplements among university students. Students were more inclined towards expensive brand supplements and had favourable opinion about them. Students had a positive attitude towards dietary supplement use however, they recommended it only upon physician’s recommendation. These findings were in line with previously reported literature.

This study used a validated questionnaire after its translation in Arabic language. The translated tool was piloted in students that enhanced understanding of the topic and improved its acceptability. This aspect was lacking in all previous researches.

The prevalence data derived from the study was very extensive. Predictive modelling applied to the data provided novel insights, i.e., prevalence, students’ attitudes and, monthly cost incurred on DS. Non-inclusion of female students could be a limitation in our study. Male and female students were segregated in different campuses with independent administration. Inclusion of female students would have helped the researchers to understand how DS use and its opinions differ gender-wise in the same population. The researchers recommend conducting the study in female population.

## Additional file

Additional file 1: Dietary supplement questionnaire (DSQ) and the Arabic version of Dietary supplement Questionnaire (DSQ-A). (PDF 267 kb)

## Abbreviations

CAGR: Compound annual growth rate
DS: Dietary supplement
DSA-A: Dietary supplement questionnaire – Arabic version
DSQ: Dietary supplement questionnaire
DV: Dependent variable
FA: Fatty acids
G6PD: Glucose 6 – phosphate dehydrogenase
IAU: Imam Abdulrahman Bin Faisal University
IV: Independent variable
KSA: Kingdom of Saudi Arabia
NHANES: National Health and Nutrition Examination Survey
SAR: Saudi Arabian riyal
SD: Standard deviation
USA: United States of America
USD: United States dollar
