# Supplementary material for: Dietary supplement use among undergraduate male students in health and non-health cluster colleges of a public-sector university in Dammam, Saudi Arabia
Source: BMC Complement Altern Med. 2018 Oct 1;18:269. doi: 10.1186/s12906-018-2332-4 (PMC6167804; doi:10.1186/s12906-018-2332-4)
Supplement: Supplementary file 1 — Dietary supplement questionnaire (DSQ) and the Arabic version of Dietary supplement Questionnaire (DSQ-A). (PDF 267 kb) [file 12906_2018_2332_MOESM1_ESM.pdf]

Questionnaire | استبيان

تقييم استخدام المكملات الغذائية بين طلاب جامعة الإمام عبد الرحمن بن فيصل، المملكة العربية السعودية

Assessing dietary supplements use among undergraduate students of health and non-health colleges at Imam Abdulrahman Bin Faisal University, Saudi Arabia

شكرًا لك لقبولك للمشاركة في هذا الاستبيان! نحن نود أن نبحث عن استخدام المكملات الغذائية لدى الطلاب. علمًا بأن هذا الاستبيان لا يسألك عن معلوماتك الشخصية ولك حرية الاختيار في المشاركة . نأمل منك ملئ هذه الاستمارة بمصداقية.

شكرًا لك !

Thank you for consenting to participate in the survey. We would like to know about the use of dietary supplements among undergraduate students. Please note that this questionnaire does not ask about your personal information and you are free to decide your participation. We hope that you would take out some time to fill this form honestly

! Thank you!

SECTION I: Demographic information		القسم الأول : معلومات اجتماعية	
Your age	○ _______years	○ ...... عام	1. العمر:
College of study	○ ________	○ كلية	2. الكلية :
Gender	○ Male<br>○ Female	○ ذكر<br>○ أنثى	3. الجنس:
Marital status	○ Single<br>○ Married<br>○ Other	○ عازب/ة<br>○ متزوج/ة<br>○ غير ذلك	4. الحالة الاجتماعية:
Number of children	○ 1 – 2 children<br>○ 3 or more children<br>○ Married without children<br>○ Not applicable to me (for single)*	○ متزوج/ة بدون أطفال<br>○ ١-٢ طفل<br>○ ٣ أو أكثر<br>○ لا ينطبق علي (عازب/ة)	5. عدد الأطفال:
Year of Study	○ Preparatory Year<br>○ 2nd Year<br>○ 3rd Year<br>○ 4th Year<br>○ 5th Year<br>○ 6th Year<br>○ 7th Year	○ السنة التحضيرية<br>○ السنة الثانية<br>○ السنة الثالثة<br>○ السنة الرابعة<br>○ السنة الخامسة<br>○ السنة السادسة<br>○ السنة السابعة	6. السنة الدراسية:
Number of siblings	○ Between 1 – 2 siblings<br>○ Between 3 – 5 siblings<br>○ Between 6 – 8 siblings<br>○ More than 8 siblings<br>○ No siblings	○ بين ١-٢<br>○ بين ٣-٥<br>○ بين ٦-٨<br>○ أكثر من ٨<br>○ لا أملك	7. عدد الأشقاء:
Resident status	○ Living with family<br>○ Living alone (University accommodation)	○ أسكن مع العائلة<br>○ مستقل	8. الحالة السكنية:
Do you suffer from any illness?	○ Yes, I suffer from ____________________<br>○ No, I do not suffer from any illness	○ نعم، أعاني من ....<br>○ لا أعاني من أي مرض	9. هل تعاني من أي مرض ؟
القسم الثاني: مدى شيوع استخدام المكملات الغذائية			
Please indicate if you use any dietary supplements in the last month?	O Daily.<br>O Weekly.<br>O Once a month.<br>O Never.<br>O I am not sure if I used dietary supplements in the last month.	○ بصورة يومية<br>○ بصورة أسبوعية<br>○ بصورة شهرية<br>○ لم أستخدم ابدا<br>○ لست متأكد اذا أستخدمتها في الشهر السابق	10. كم مرة أستخدمت فيها أحد أنواع المكملات الغذائية خلال الشهر السابق ؟
القسم الثالث: تكاليف المكملات الغذائية			
How much your dietary supplement cost you in the last month? (Not applicable if not used)*	_________ SAR	قد كلفتني .... ريال<br>كم كلفتك المكملات الغذائية في الشهر السابق؟<br>* (لا ينطبق إذا لا تستخدم المكملات الغذائية) *	11. كم كلفتك المكملات الغذائية في الشهر السابق (لا ينطق اذا كنت ممن لا يستخدمون المكملات الغذائية)

Which dietary supplement you used last month?	○ Generic<br>○ Brand<br>○ Both<br>○ I don’t know<br>○ Not applicable to me as I did not use	○ مكملات محلية الصنع<br>○ مكملات عالمية الصنع<br>○ الاثنين<br>○ لا اعلم<br>○ لا ينطبق علي لانني لست ممن يستخدم المكملات الغذائية	12. ما نوع المكملات الغذائية التي استخدمتها خلال الشهر الماضي؟
			القسم الرابع: الآثار الضارة للمكملات الغذائية
In the last month did you experience any side effects that were related to your dietary supplements use?	○ Yes, I suffered from<br>○ I suffered from adverse event but not sure if it was related to dietary supplement use<br>○ No, I did not suffer from any adverse effect<br>○ Not applicable to me as I did not use any dietary supplement	○ نعم، عانيت من ....<br>○ لقد عانيت من عرض جانبي ولكن لست متأكدا ما إذا كان يتعلق باستخدامي إلى المكملات الغذائية<br>○ لا، لم اعاني من اي عرض جانبي<br>○ لا ينطبق علي لانني لست ممن يستخدم المكملات الغذائية	13. في الشهر الماضي هل عانيت من أي عرض جانبي يتعلق بالمكملات الغذائية الخاصة بك ؟
			القسم الخامس: اسباب استخدام المكملات الغذائية
Reason for taking dietary supplements?	(You may select more than one)*<br>○ General health and well being<br>○ Boost immunity<br>○ Weight gain<br>○ Doctor's recommendation<br>○ Enhance memory<br>○ Increase performance/sports<br>○ Increase endurance/ body building<br>○ Joint care<br>○ I have a poor diet<br>○ Energy source<br>○ Other reason_________________________	(بإمكانك اختيار أكثر من أختيار)<br>○ الصحة العامة<br>○ تعزيز المناعة<br>○ زيادة الوزن<br>○ تعليمات الطبيب<br>○ تعزيز الذاكرة<br>○ رفع الأداء الرياضي<br>○ بناء الأجسام<br>○ العناية بالمفاصل<br>○ نظامي الغذائي غير كافي<br>○ مصدر طاقة<br>○ اخرى (إذكرها)	14. ما هو سبب استعمالك إلى المكملات الغذائية ؟
Which of the following dietary supplement you used in particular?	**2.** (You may select more than one)*<br>○ I did not use<br>○ Multivitamins<br>○ Ginseng<br>○ Gingko biloba<br>○ Glucosamine / omega 3 fatty acids<br>○ Whey protein<br>○ Evening Primrose oil<br>○ Other______________________	(بإمكانك اختيار أكثر من واحد)<br>○ انا لا استخدم<br>○ فيتامينات<br>○ الجنسنغ<br>○ الجنكة (جنكو بيلوبا)<br>○ Glucosamine / Omega 3<br>○ Whey protein<br>○ Evening Primrose oil<br>○ اخرى (إذكرها)	15. اي مما يلي من المكملات الغذائية تستخدمه بالتحديد ؟
			القسم السادس: آراء واعتقادات حول المكملات الغذائية
Is dietary supplement good for health?	○ Agree<br>○ I don't know<br>○ Disagree	○ أتفق<br>○ لا أعلم<br>○ لا أتفق	16. هل تتفق أن المكملات الغذائية مفيدة للصحة ؟
Do you encourage the use of dietary supplements?	○ Yes, I always recommend<br>○ Yes, only when doctors recommend<br>○ Not at all	○ نعم، أنا أستحسنها<br>○ نعم، بشرط أن يستحسنها الطبيب<br>○ لا أبدا	17. هل تشجع على استخدام المكملات الغذائية ؟

Your opinion about dietary supplements	○ Necessary for all ages<br>○ They are harmless<br>○ Regular use of Dietary supplements prevents chronic diseases<br>○ Dietary supplements may prevent cancer	○ ضرورية لكل الأعمار<br>○ غير مضرة بالصحة<br>○ الاستخدام المنتظم للمكملات الغذائية يقي من الأمراض المزمنة<br>○ المكملات الغذائية قد تقي من مرض السرطان	18. ما هو اعتقادك حول المكملات الغذائية ؟
How do you rate your experience with dietary supplement use?	WORST ← 0 – 1 – 2 – 3 – 4 – 5 → BEST	سيئة ← 0 – 1 – 2 – 3 – 4 – 5 → ممتازة	19. ماهو تقييمك الى تجربتك مع المكملات الغذائية ؟
What is your major source of information regarding dietary supplement use?	○ Friends, family, relatives<br>○ Doctors and pharmacists<br>○ Internet<br>○ Newspaper and magazines<br>○ Other ____________________	○ الأصدقاء ، العائلة ، الأقارب<br>○ الأطباء والصيادلة<br>○ الإنترنت<br>○ الجرائد والمجلات<br>○ أخرى ____________________	20. ما هو المصدر الرئيسي للمعلومات المتعلقة باستخدام المكملات الغذائية؟

شكرا لك
☺
